# Sensitivity Enhancement of FBG-Based Strain Sensor

**DOI:** 10.3390/s18051607

**Published:** 2018-05-17

**Authors:** Ruiya Li, Yiyang Chen, Yuegang Tan, Zude Zhou, Tianliang Li, Jian Mao

**Affiliations:** 1 School of Mechanical and Electronic Engineering, Wuhan University of Technology, Wuhan 430070, China; liruiya@whut.edu.cn (R.L.); cyy5284@whut.edu.cn (Y.C.); zudezhou@whut.edu.cn (Z.Z.); maojiancool@whut.edu.cn (J.M.); 2 School of Engineering, University of Birmingham, Birmingham B15 2TT, UK; 3 Department of Biomedical Engineering, National University of Singapore, Singapore 117576, Singapore; bieliti@nus.edu.sg

**Keywords:** fiber Bragg grating, strain, sensitivity enhancement, lever structure

## Abstract

A novel fiber Bragg grating (FBG)-based strain sensor with a high-sensitivity is presented in this paper. The proposed FBG-based strain sensor enhances sensitivity by pasting the FBG on a substrate with a lever structure. This typical mechanical configuration mechanically amplifies the strain of the FBG to enhance overall sensitivity. As this mechanical configuration has a high stiffness, the proposed sensor can achieve a high resonant frequency and a wide dynamic working range. The sensing principle is presented, and the corresponding theoretical model is derived and validated. Experimental results demonstrate that the developed FBG-based strain sensor achieves an enhanced strain sensitivity of 6.2 pm/με, which is consistent with the theoretical analysis result. The strain sensitivity of the developed sensor is 5.2 times of the strain sensitivity of a bare fiber Bragg grating strain sensor. The dynamic characteristics of this sensor are investigated through the finite element method (FEM) and experimental tests. The developed sensor exhibits an excellent strain-sensitivity-enhancing property in a wide frequency range. The proposed high-sensitivity FBG-based strain sensor can be used for small-amplitude micro-strain measurement in harsh industrial environments.

## 1. Introduction

The strain is one of the most important monitored physical parameters in the structural health monitoring (SHM) of modern mechanical equipment [1,2,3]. In some cases, SHM requires a high strain sensitivity to the applied strain sensors. For instance, detecting strain shifts caused by fatigue crack is often problematic because of the very low strain amplitudes, which require a strain sensor with a sub-microstrain resolution. Conventionally, strain measurements in this kind of situation rely on the use of resistive strain gauges with a resolution of about 0.5 με, which are commercially available and standardized. In terms of frequency response, resistive strain gauges have a wide working range (max. 1 kHz~50 kHz), which can meet most requirements in SHM. However, the measured signals from traditional electrical strain sensors are easily contaminated by electromagnetic interference (EMI) in a harsh industrial environment, which deteriorates the performance of an SHM system [4]. Fiber Bragg grating (FBG)-based strain sensors exhibit advantages such as immunity to EMI, resistance to corrosion, and multiple measuring points in one optical fiber, which have attracted a great deal of attention and have been widely investigated in the SHM research field recently [5,6,7,8,9,10,11,12].

Commonly, bare FBG strain sensors are directly bound on the surfaces of monitored structures using adhesives to detect strain shifts [13,14,15,16] or are embedded in some composite material to form smart structures [17,18]. The strain sensitivity of directly-pasted or embedded bare FBG sensors is 1.21 pm/με, in theory [19]. As the resolution and precision of a dynamic FBG integrator are 1 pm and ±5 pm, the resolution and precision of a bare FBG strain sensor are about 0.8 με and ±4 με, which can meet most engineering applications. However, in some cases, the strain to be tested is at a very low level (e.g., 1~10 με, 10~20 με), the 1.21 pm/με sensitivity of the bare FBG cannot be qualified for the accurate detection of this small-amplitude strain due to the noise of the FBG interrogator. Therefore, a FBG strain sensor with a higher sensitivity and accuracy is urgently needed. Some researchers bonded the two ends of a FBG with two high-stiffness tubes, respectively, and then bonded the outer ends of the two tubes to the structure surface to get a relatively higher strain sensitivity. For instance, Ren et al. [20] designed a FBG strain sensor that can achieve a sensitivity of 2.053 pm/με through packaging the FBG using two gripper tubes. Li et al. [21] developed a FBG strain sensor with a sensitivity of 2.52 pm/με in the same way, for the long-term structural health monitoring system of a highway bridge. The above-mentioned sensors enhance strain sensitivity by centralizing the continuous strain of a long region on the tested structure to the short FBG area. However, the distance between the two bonded points of the sensor increases the whole volume of the sensor (254 mm length; in Reference [21]). Additionally, some other researchers utilized substrates with flexure hinges to enlarge the strain at the FBG area. Zhang et al. [22] presented a diamond-frame packaged FBG strain sensor, which was temperature-insensitive and the strain sensitivity was enhanced to 1.814 pm/με. Guo et al. [23] used a substrate with flexure hinges to improve the strain sensitivity of a FBG-based sensor. The experimental results showed that this sensor’s sensitivity could reach 3.357 pm/με. These sensors can also achieve a higher strain sensitivity compared with directly-pasted bare FBG sensors. However, they are fragile and easy to be broken due to the thin flexure hinges. Nawrot et al. [24,25] proposed a mechanical transducer that amplifies the strain applied to FBGs. The amplification factor is larger than 30, which makes small-amplitude strains easier to be detected. The outer dimensions of this sensor are 380 × 105 mm^2^. It can be applied to large engineering structures (e.g., bridges, heavy-duty gantry cranes, etc.), but it is too large to be used in industrial equipment in regular sizes. The dynamic working range is an important parameter of a strain sensor. However, References [20,21,22,23] do not provide this property as they are used for static or quasi-static strain measurements. Through the finite element method, Nawrot et al. [24,25] analyzed the resonant frequency of their sensor. When the thickness of their sensor changes as 3 mm, 5 mm, 7 mm, and 9 mm, the corresponding resonant frequencies are 148 Hz, 244 Hz, 338 Hz, and 409 Hz, respectively. The working frequency bandwidth of the sensor is not wide enough for high-frequency dynamic strain measurements in mechanical equipment.

This paper presents a sensitivity-enhanced FBG strain sensor based on a substrate with a lever structure. The proposed sensor has a smaller and simpler structure and a much higher resonant frequency than the sensor in References [24,25]. Firstly, the structure and sensitization model of the sensor is introduced in Section 2. Then, in Section 3, the simulation results using the finite element method (FEM) are discussed. Finally, the experimental study of the sensing properties of the developed sensor is explained in Section 4. The strain sensitivity of the FBG strain sensor can be effectively improved by using the lever principle. Its sensitivity is over five times larger than the directly-pasted bare FBG. Adjusting the size of the lever structure can easily regulate the sensitivity and precision of the FBG strain sensor. The designed sensor has the advantages of a simple and compact structure, high strain sensitivity in a wide frequency range, convenient installation, and high consistency and reliability. The developed sensor can assist in small-amplitude micro-strain measurements in a harsh industrial environment.

## 2. Sensitization Model of the Sensor

### 2.1. The Principle of Strain Sensing of FBG

FBG consists of a periodic modulation of the refraction along the fiber core. When a broadband light propagates along the optical fiber core to the fiber Bragg grating, the light (with a particular wavelength which satisfies the Bragg interference condition) is reflected back while the rest of the light is transmitted with a small attenuation. The particular wavelength of the reflected light is regarded as the Bragg wavelength, and is expressed by the following equation:

(1) $$\lambda_{B}=2n_{eff}\Lambda$$

where *λ_B_* is the Bragg wavelength of the FBG, *n_eff_* is the effective refractive index of the fiber core at the free space center wavelength, and Λ is the grating periodicity of the FBG. An FBG is very sensitive to strain changes. The shifts in strain will cause changes of *n_eff_* or Λ, and lead to the shifts of *λ_B_*. Hence, by monitoring the Bragg wavelength shift, the value of the strain can be determined. The wavelength variation response to the strain *ε*_FBG_ can be given by:

(2) $$\frac{\Delta \lambda_{B}}{\lambda_{B}}=(1-p_{e})\varepsilon_{\mathrm{FBG}}$$

where *p_e_* = 0.22 is the effective photo-elastic coefficient.

### 2.2. Structure and Strain Amplification Mechanism

**Figure 1:**
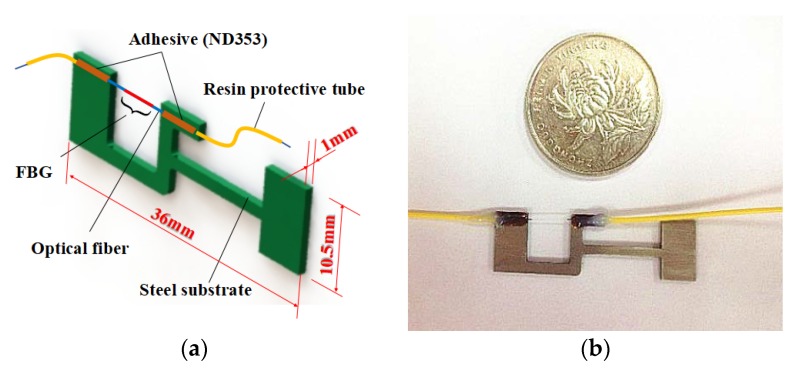
The designed FBG strain sensor: (**a**) The structure of the designed sensor; (**b**) real image of the designed sensor.

**Figure 2:**
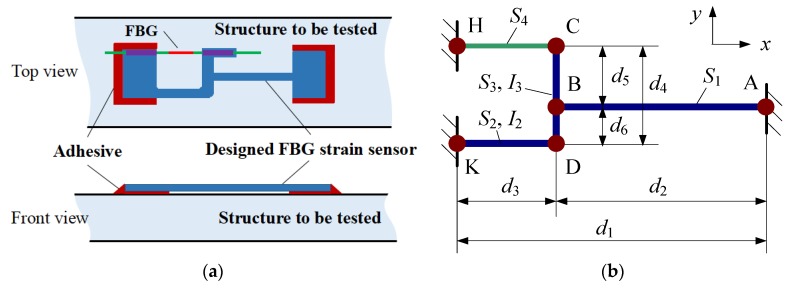
Fixing method and simplified sensing model of the designed sensor: (**a**) Fixing method; (**b**) simplified model.

The structure of the developed fiber Bragg grating strain sensor is shown in Figure 1. It is mainly composed of a bare FBG sensor and an elastic substrate with a lever structure. The double ends of the bare FBGs are fixed to the elastic substrate using adhesive (ND353). It is noteworthy that pre-tension should be applied to the bare FBG when fixing it on the substrate to ensure the FBG can effectively detect compressive strain. The tail fiber of the bare FBG is packaged in plastic protective sleeves. The outer dimensions of this sensor are 36 × 10.5 mm^2^, and the thickness of the sensor is 1 mm. The total weight of the sensor is about 1.5 g. The substrate is made of stainless steel 304. As its stiffness is high, it can improve the dynamic working range of the sensor. On the other hand, this sensor cannot be used to detect strain on structures made of a low-stiffness material (e.g., rubber) since it may affect the deformation of the tested structure. The coating layer of the FBG was removed since it weakens the strain transfer effect. The physical parameters of the main components of the designed sensor are illustrated in Table 1. The fixing method of the developed FBG strain sensor is shown in Figure 2a. The double ends of the substrate in the designed FBG strain sensor were bonded to the structure to be tested using adhesive. Reference [20] demonstrated that the double-ends adhesive fixed method can achieve commendable strain transfer effects. Figure 2b shows the simplified model of the designed sensor. The structure of the substrate can be simplified by the rod connections, taking the optical fiber with a FBG into consideration (rod CH).

**Figure 3:**
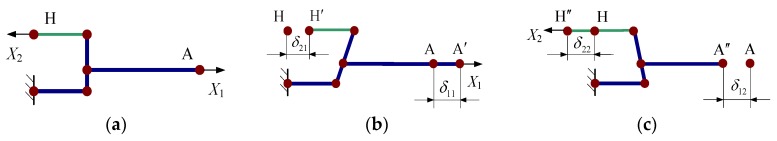
Displacement analysis of the secondary hyperstatic structure: (**a**) Replacing the constraint conditions of point A and H with unit forces; (**b**) virtual displacement caused by *X*_1_; (**c**) virtual displacement caused by *X*_2_

When the structure surface to be tested generates a strain *ε*, a relative displacement Δ*d* is generated between point A and point H (or K) in the *x*-axis direction. Assuming the displacements of point H, K is zero, the displacement at point A will be Δ*d* in the *x*-axis direction. Correspondingly, we can get that *ε* = Δ*d*/*d*_1_. At the same time, forces and bending moments will be generated at point A, H, and K. Under small deformation conditions, the forces in the *y*-axis direction and bending moments at point A and point H are very small and not taken into account in the analytical study. Therefore, the whole structure can be simplified as a secondary hyperstatic structure. Replacing the constraint conditions of point A and H with the unit forces *X*_1_ and *X*_2_, respectively (as shown in Figure 3a), based on the law of Virtual Work and Unit Load Method, displacement *δ*_11_ and *δ*_12_ will be generated, respectively, at point A when *X*_1_ and *X*_2_ act independently. In the same way, deformations *δ*_21_ and *δ*_22_ will be generated, respectively, at point H when *X*_1_ and *X*_2_ act independently (as shown in Figure 3b,c). According to the deformation compatibility condition, the regular equation can be obtained:

(3) $$\left[\begin{array}{cc} \delta_{11} & \delta_{12}\\ \delta_{21} & \delta_{22} \end{array}\right]\cdot \left[\begin{array}{c} X_{1}\\ X_{2} \end{array}\right]=\left[\begin{array}{c} \Delta d\\ 0 \end{array}\right]$$

According to the law of Virtual Work in the theory of material mechanics. When a material obeys Hooke’s law, and in the case of small deformations, there is a linear relationship between the virtual displacement and the applied load on the structure, which can be expressed by More’s integral as follows:

(4) $$\delta =\int_{l}\frac{F_{N}(x)f_{N}(x)}{ES}dx+\int_{l}\frac{M(x)m(x)}{EI}dx+\int_{l}\frac{T(x)t(x)}{GI_{p}}dx$$

where *δ* represents the virtual displacement, *F*_N_(*x*), *M*(*x*), and *T*(*x*) are the internal force, bending moment, torque caused by the actual load, respectively. *f*_N_(*x*), *m*(*x*), and *t*(*x*) are the internal force, bending moment, torque caused by the unit load, respectively. *E* and *G* represent the elastic modulus and shear modulus of the structure respectively. *S* is the cross-sectional area. *I* and *I*_p_ are the area moment of inertia and the torsional moment of inertia respectively. The values of virtual displacements *δ*_11_, *δ*_12_, *δ*_21_ and *δ*_22_ can be calculated using the More’s integral:

(5) $$\left\{ \begin{array}{l} \delta_{11}=\frac{d_{3}}{ES_{2}}+\frac{d_{2}}{ES_{1}}+\frac{d_{6}^{2}\cdot d_{3}}{EI_{2}}+\frac{d_{6}^{3}}{3EI_{3}}\\ \delta_{22}=\frac{d_{3}}{ES_{2}}+\frac{d_{3}}{E_{\mathrm{FBG}}S_{4}}+\frac{d_{4}^{3}}{3EI_{3}}+\frac{d_{4}^{2}\cdot d_{3}}{EI_{3}}\\ \delta_{12}=\delta_{21}=-\left(\frac{d_{3}}{ES_{2}}+\frac{d_{6}^{2}\cdot (3d_{5}+d_{6})}{6EI_{3}}+\frac{d_{6}^{2}\cdot d_{3}}{EI_{2}}\right) \end{array}\right.$$

The values of the related parameters in Figure 2b and Equation (5) are shown in Table 1 and Table 2. Substituting Equation (5) into Equation (3), we can obtain that *X*_1_ = 1.5Δ*d* and *X*_2_ = 0.2Δ*d*. The deformation of FBG (rod CH) Δ*d*_FBG_ can be expressed as Δ*d*_FBG_ = (*X*_2_*d*_3_)/(*E*_FBG_*S*_4_) = 2.16Δ*d*. Thus, the sensitivity amplification factor *q* of the designed sensor can be calculated as following:

(6) $$q=\frac{\varepsilon_{\mathrm{FBG}}}{\varepsilon }=\frac{\Delta d_{FBG}/d_{3}}{\Delta d/d_{1}}=\frac{\Delta d_{\mathrm{FBG}}\cdot d_{1}}{\Delta d\cdot d_{3}}$$

where *ε*_FBG_ is the strain of the silica fiber in the FBG area. Substituting the values of the parameters in Table 2 into Equation (6), we can get that 5.7 is the theoretical value of sensitivity amplification factor *q*. According to the principle of fiber Bragg grating sensing, the wavelength shift Δ*λ_B_* of the FBG can be obtained as follows:

(7) $$\frac{\Delta \lambda_{B}}{\lambda_{B}}=(1-p_{e})q\varepsilon$$

(8) $$k=\frac{\Delta \lambda_{B}}{\varepsilon }=\lambda_{B}(1-p_{e})q$$

When *λ_B_* = 1550 nm, the theoretical strain sensitivity *k* of the developed FBG strain sensor is 6.9 pm/με.

## 3. Simulation Analysis

### 3.1. Static Analysis

**Figure 4:**
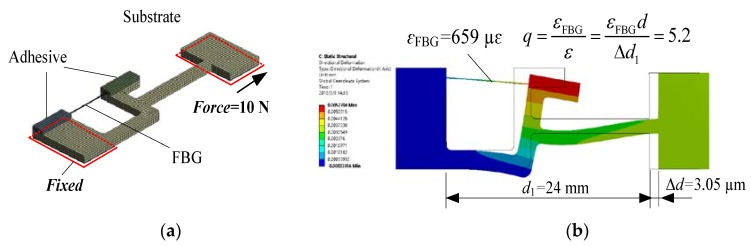
Static structural analysis based on FEM: (**a**) FEM model; (**b**) FEM analysis results.

Theoretical analysis by FEM was carried out using ANSYS software to verify the feasibility of the proposed method and structure. The physical properties of the material involved in the designed sensor were set according to the details in Table 1. As shown in Figure 4a, one end of the substrate of the sensor was fixed, and a 10 N force was implemented on the other end of the substrate. Figure 4b exhibits that the substrate generated a deformation of Δ*d* = 3.05 μm at this load, which reflects that the strain to be tested is *ε* = Δ*d*/*d*_1_ = 127.08 με. Under the same situation, the strain *ε*_FBG_ of the FBG is 659 με. The finite element analysis results demonstrate that strain sensitivity amplification factor *q* is 5.2, which is consistent with the theoretical result (5.7). The sensitivity amplification factor calculated using the FEM is a little smaller than the value obtained by the theoretical calculations of the simplified model. The main reasons involve:

- The lever structure is simplified as a rod structure in the theoretical calculation.
- The theoretical calculation parameters are based on central sizes of the simplified rods.
- The characteristics of the adhesive are not considered in the theoretical calculations, while the adhesive thickness between the substrate of the developed FBG strain sensor and the specimen reduces the strain transform coefficient in the FEM analysis.

### 3.2. Dynamic Characteristics Analysis

**Figure 5:**
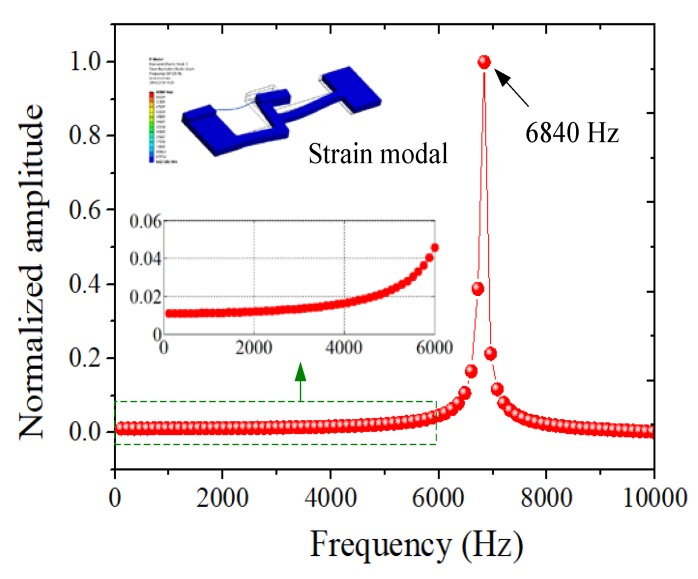
The harmonic response of the ANSYS simulation.

Strain transducers are generally required to possess good dynamic performance to accurately obtain the dynamic characteristics of a structure to be measured. The dynamic characteristics of the designed sensor were analyzed via modal analysis and harmonic response analysis using ANSYS. The modal analysis results show that the resonant frequency of the designed sensor is 6813.5 Hz. Figure 5 shows the normalized strain amplitude of the FBG when the substrate is under a harmonic force with a 10-N amplitude and 0~10,000 Hz frequency range (the interval is 20 Hz). The maximum strain of the FBG occurs when the frequency is near the resonant frequency. The designed sensor has a flat response when the frequencies are less than 6000 Hz.

## 4. Experimental Study of the Sensing Properties

### 4.1. Static Properties

**Figure 6:**
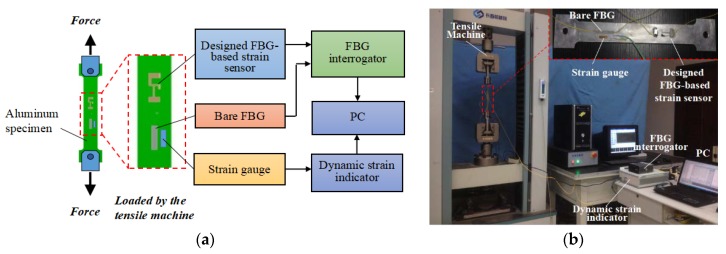
Experimental setup: (**a**) Schematic diagram; (**b**) real images.

Figure 6 shows the schematic of the experimental setup and instruments. The designed FBG-based strain sensor, the bare FBG sensor, and the resistance strain gauge were bonded on the surface of an aluminum specimen in the same sensing direction. The aluminum specimen was installed on the tensile testing machine, which accurately controlled the tensile force applied to the specimen. An FBG interrogator (GAUSSIAN OPTICS) with a 1 pm resolution and a 1 Hz sampling rate was used to detect the central wavelength shifts of the designed FBG-based strain sensor and the bare FBG sensor. A high-resolution resistance strain indicator (TST5912) was utilized to demodulate the signals of the resistance strain gauge. During the experiment, the tensile machine was controlled to load and unload in the range of 0–2000 N with an interval of 200 N. At every load/unload step, the test signals from the FBG interrogator and resistance stain indicator were collected. As the test time was relatively short, room temperature could be considered constant.

**Figure 7:**
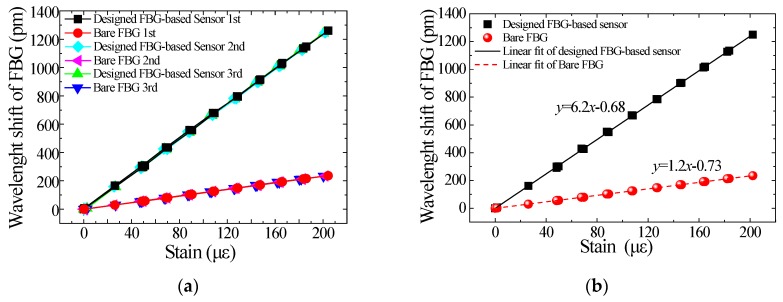
Repeatability and linearity of the FBG strain sensor: (**a**) Repeatability experiment; (**b**) linear fitting.

Here, we assume that the strain transform coefficients from the surface of the aluminum specimen to the strain gauge, bare FBG, and the designed sensor were all 100%. The strain detected by the strain gauges was regarded as the real strain on the specimen surface. Figure 7a lists the wavelength shifts of the designed sensor and the bare FBG in the experiments to demonstrate the repeatability of the FBG strain sensor. The repeatability error of the designed sensor is less than 0.5%. Figure 7b shows the linear fitting results of repeated experimental data. The strain sensitivity of the designed sensor is 6.2 pm/με and the linear correlation coefficient is 0.99986. The actual strain amplification factor of the designed FBG strain sensor is 5.2, which matches the FEM result. As the resolution and precision of the FBG integrator are 1 pm and ±5 pm respectively, the resolution and precision of the developed sensor are 0.16 με and ±0.80 με respectively.

### 4.2. Dynamic Properties

**Figure 8:**
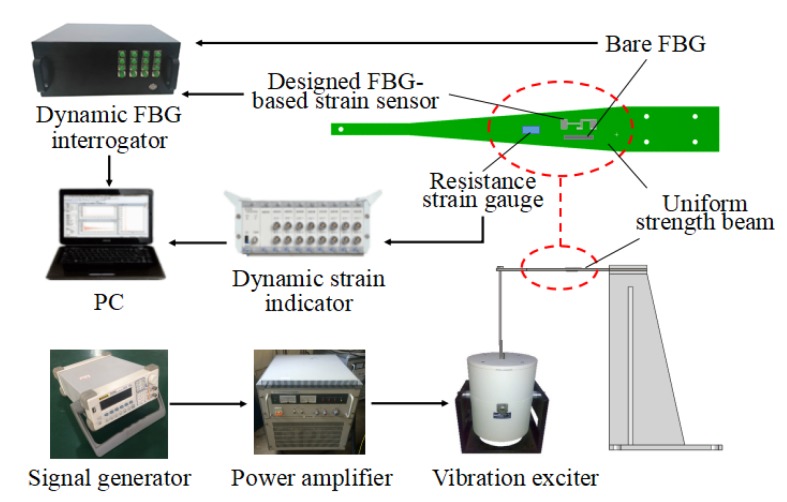
Schematic diagram of dynamic properties experiment.

In order to investigate the dynamic capabilities of the developed sensor, a dynamic experimental setup was established (as shown in Figure 8). The designed sensor was fixed on the uniform strength beam which was excited by a vibration exciter. In contrast, a dynamic strain gauge and a bare FBG were also fixed on the same side of the uniform strength beam and in the same sensing direction. The signals of the designed sensor and the bare FBG were collected by the FBG interrogator with a sampling rate of 2 kHz. The signal of the resistance stain gauge was recorded by a dynamic strain indicator (TST5912). The driving signal of the vibration exciter was controlled by the signal generator and power amplifier.

**Figure 9:**
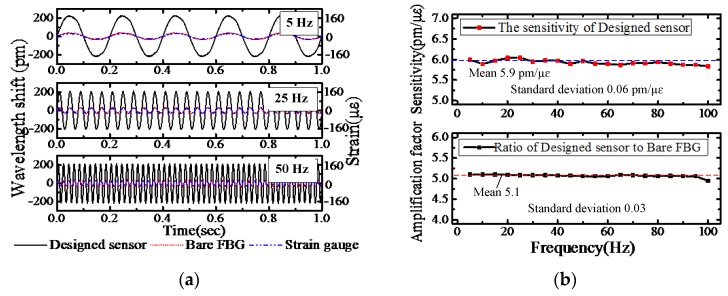
The response of FBG stain sensor, bare sensor and strain gauge under 5–100 Hz excitation frequency: (**a**) Dynamic strain measurement; (**b**) the sensitivity and amplification factor.

The dynamic response of the FBG strain sensor was tested with the excitation frequencies varied in the range of 5 Hz to 100 Hz with an interval of 5 Hz. As the power of the vibration is too low to exert a greater force on the cantilever beam when the frequency is high, the highest excitation frequency was set to 100 Hz. The test results were compared with the bare FBG and the strain gauge. It is worthwhile pointing out that as the designed sensor has a thickness of 1 mm. It is unfair to compare the testing results of bare FBG and the designed sensor directly when they are utilized to test the bending strain on the uniform strength beam. The thickness of the uniform strength beam is 8 mm, therefore, the distance between the bare FBG and the neutral layer of the uniform strength beam is 4 mm while the distance between the FBG on the designed sensor and the neutral layer of the uniform strength beam is 5 mm. The detected wavelength shifts of the designed sensor were divided by *t* = 5/4 = 1.25 to eliminate the sensitization effect caused by the thickness of the substrate of the designed sensor. As shown in Figure 9, the strain sensitivity and strain amplification factor of the designed sensor remains consistent with the static test results at a frequency range from 5 Hz to 100 Hz. The mean values of the strain sensitivity and strain amplification factor are 5.9 pm/με and 5.1 respectively, considering the thickness of the designed FBG sensor. The maximum errors are 2.7% and 2.4%, respectively. The standard deviations of the tested strain sensitivity and strain amplification factor are 0.06 pm/με and 0.03, respectively.

**Figure 10:**
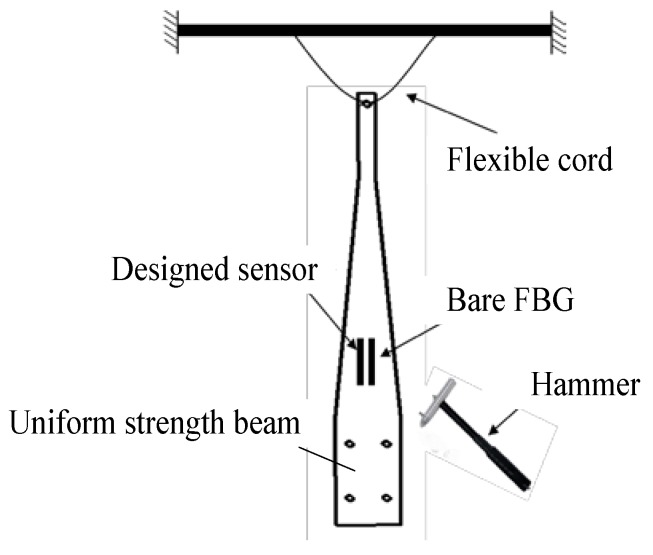
Schematic diagram of knocking experiment.

Vibration tests—by knocking on the cantilever beam—were carried out to get the response of the designed FBG strain sensor at higher frequencies. The cantilever beam was hung using elastic rope to make sure it was in a free foundation, as shown in Figure 10. The signals of the designed FBG strain sensor and bare FBG were collected—by knocking the cantilever beam with a hammer—by the FBG interrogator (sampling rate: 2 kHz).

**Figure 11:**
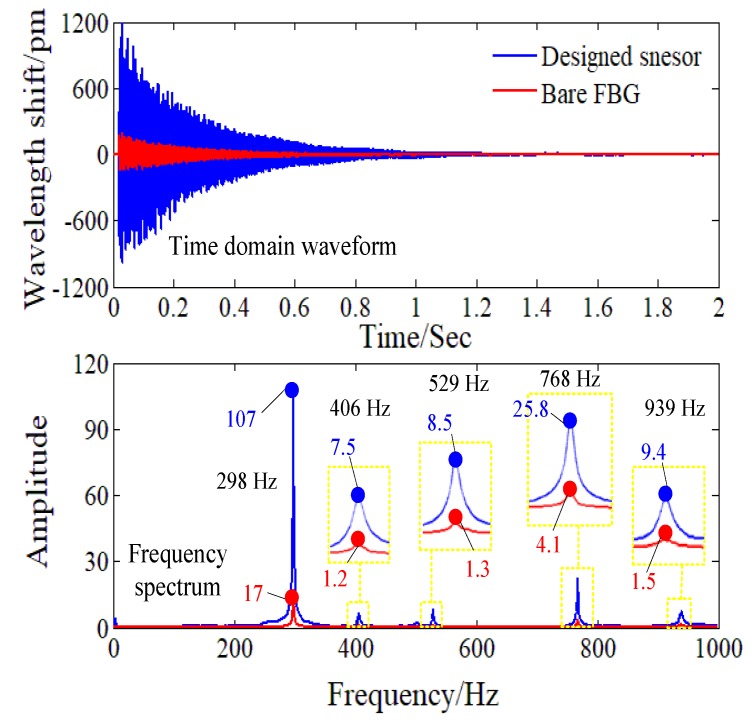
Time domain waveform and the frequency spectrum of the bare FBG and the designed sensor in the knocking experiment.

From the time domain waveform in Figure 11, it can be seen clearly that the designed sensor is more sensitive than the bare FBG in the knocking experiment. To quantitatively compare the strain sensitivity of the designed FBG sensor and the bare FBG, we obtained the frequency spectrum of the testing results via fast Fourier transform (FFT). According to the laws of the sampling, as the sampling rate of the FBG interrogator is 2 kHz, the frequency spectrum from 0 to 1 kHz can be analyzed. As shown in Figure 11, the frequency spectrum obtained from the designed sensor’s testing data and the frequency spectrum obtained from the bare FBG’s testing data show a high concordance in spectral distributions. Both the designed FBG strain sensor and the bare FBG can detect the first-order eigen-frequency 298 Hz and the second-order eigen-frequency 768 Hz of the uniform strength beam, which is consistent with the modal analysis results of 280.1 Hz and 728.7 Hz, respectively, by FEM. For the dynamical strain in 298 Hz and 768 Hz, the sensitivity amplification factor *q* of the designed FBG sensor are 107/(17 × 1.25) = 5.0 and 25.8/(4.1 × 1.25) = 5.0, respectively, considering the thickness of the substrate of the designed sensor. Additionally, in the knocking experiment, dynamic strain signals in some other frequencies (406 Hz, 529 Hz, and 939 Hz) have been stimulated. For dynamical strain in these frequencies, the sensitivity amplification factor *q* of the designed FBG sensor are 7.5/(1.2 × 1.25) = 5.0, 8.5/(1.3 × 1.25) = 5.2, 9.4/(1.5 × 1.25) = 5.0, respectively. Therefore, the designed sensor keeps a constant sensitivity amplification factor in a wide frequency range.

Comparing to existing FBG-based strain sensors in References [20,21,22,23,24,25], the particular mechanical configuration in this paper brings lots of advantages to the designed sensor. Firstly, the proposed sensor has a small and compact structure, which make it possible to apply it in regular-sized mechanical equipment. Secondly, the structural design of the sensor is very simple, which makes it easier to manufacture. The simple structure can enhance the reliability of the sensor in engineering applications. Thirdly, the particular mechanical configuration in the designed sensor can easily amplify the strain of FBG to enhance the overall sensitivity based on the lever principle. Through changing the sizes and materials of the mechanical configuration, the strain amplification factor of the mechanical configuration can be controlled to transfer more or less strain to the FBG. Consequently, the measurement range and sensitivity can be easily adjusted to adapt to different measurement demands. Finally, the developed sensor possesses good dynamic response characteristics and can be qualified for dynamic strain measurements in a wide frequency range with a constant high strain sensitivity.

## 5. Conclusions

A sensitivity enhancing method for FBG-based strain sensor is proposed in this paper. A sensitivity enhanced FBG-based strain sensor was designed and realized. This sensor mainly consists of an FBG and an elastic substrate with a lever structure which mechanically amplifies the strain at FBG area. The strain sensing model of the designed sensor was analyzed by material mechanics theory and was verified through FEM and experimental test. The designed sensor has a strain sensitivity of 6.2 pm/με, which is as 5.2 times of the bare FBG sensor. The designed sensor possesses a good linearity and a low repeatability error. In addition, as the lever structure of the designed sensor has a high stiffness, the proposed sensor can achieve a high resonant frequency, and its dynamic test range is wide. Due to its high strain sensitivity, the designed sensor has a wide application prospects for the small-amplitude micro-strain measurements in harsh industrial environments.

## Figures and Tables

**Table 1:** The physical parameters of the sensor.

Structure	Material	Elastic Modulus	Poisson Ratio
Substrate	Stainless steel 304	*E* = 200 Gpa	ν = 0.3
FBG	Silica	*E*_FBG_ = 74.52 Gpa	ν_FBG_ = 0.17
Adhesive	Epoxy resin	*E*_a_ *=* 3.0 Gpa	ν_a_ = 0.38

**Table 2:** The values of parameters in the simplified sensing model.

Parameters	Values	Parameters	Values
*d*_1_	24 mm	*S*_1_	1.5 mm^2^
*d*_2_	15 mm	*S*_2_	2 mm^2^
*d*_3_	9 mm	*S*_3_	2 mm^2^
*d*_4_	8.5 mm	*S*_4_	0.012272 mm^2^
*d*_5_	5 mm	*I*_2_	2/3 mm^4^
*d*_6_	3.5 mm	*I*_3_	2/3 mm^4^
